# Trained Immunity in Autoimmunity: Friend, Foe, or Therapeutic Target?

**DOI:** 10.3390/biomedicines14030526

**Published:** 2026-02-26

**Authors:** Hugo Abreu, Davide Raineri, Annalisa Chiocchetti, Giuseppe Cappellano

**Affiliations:** 1 Department of Health Sciences, Interdisciplinary Research Center of Autoimmune Diseases-IRCAD, University of Eastern Piedmont, 28100 Novara, Italy; davide.raineri@uniupo.it (D.R.); annalisa.chiocchetti@med.uniupo.it (A.C.); giuseppe.cappellano@med.uniupo.it (G.C.); 2 Center for Translational Research on Autoimmune and Allergic Diseases, University of Eastern Piedmont, 28100 Novara, Italy

**Keywords:** trained immunity, autoimmune diseases, innate immune system, immunological memory

## Abstract

For decades, immunology has followed a clear paradigm: immunological memory resides only within the adaptive immunity, as a unique property of lymphocytes giving the host the ability to recognize specific antigens and offer long-term protection. However, this raises an important question: how valid is this belief in light of new evidence? The discovery of trained immunity shows that innate immune cells can also develop lasting functional changes. This finding prompts a profound reconsideration of the traditional framework. Trained immunity is a functional reprogramming of the innate immune cells driven by long-term epigenetic and metabolic reprogramming, resulting in enhanced responses upon subsequent exposure to the same pathogen or even to unrelated stimuli. The presence of pattern recognition receptors (PRRs) on innate immune cells already suggested a certain level of specificity in this compartment thanks to the engagement of a PRR by a pathogen-associated molecular pattern (PAMP) inducing memory-like properties in the responding cell. While such partial specificity can enhance protection, it may also amplify aberrant inflammatory circuits, thereby contributing to the initiation or worsening of autoimmune and chronic inflammatory diseases. This dual nature of trained immunity raises important questions for the field: is trained immunity ultimately harmful or beneficial in autoimmunity, and can its mechanisms be harnessed therapeutically rather than pathologically? The present Perspective will address these issues by examining recent findings that reveal the specificity, pathogenic potential, and translational opportunities in given examples of autoimmune diseases (ADs).

## 1. Revisiting the Classical Paradigm of Immunological Memory

Immunologic memory is the ability of the immune system to respond more rapidly and effectively upon re-exposure to a pathogen and involves long-lived, clonally expanded, antigen-specific lymphocytes. Immune memory is a key feature of the adaptive immune system, generated following an initial immune response to an antigen, referred to as the primary response, with a memory immune response persisting long after the antigen is cleared [1].

During a primary response, antigen-presenting cells (APCs) activate antigen-specific T cells by presenting pathogen peptides on major histocompatibility complex (MHC). T cells expand, secrete interleukin (IL)-2, become effectors, and migrate to clear infection. Follicular helper T cells support the activation of B cells and the formation of germinal centers that produce antibodies that neutralize pathogens. After clearance, most effector cells die, while memory T and B cells and long-lived plasma cells remain for long-term protection [2].

Conversely, innate immune responses were traditionally thought to be nonspecific and without memory since innate immune cells engage pathogens in the first hours and days of an infection [3]. These cells were believed to be unable to adapt after an infection and build immunologic memory, mainly because adaptive memory relies on clonality: when a B or T cell recognizes a specific antigen, it clonally expands, making thousands of identical memory cells [4]. Innate immune cells cannot clonally expand in an antigen-specific way, so they do not rely on clonal selection. How does innate immune memory actually develop following a primary stimulus?

The majority of innate immune cells express pattern recognition receptors (PRRs) that recognize specific components of encountered pathogens [5]. Engagement of PRRs does more than initiate an acute inflammatory response, by driving durable epigenetic and metabolic rewiring within innate cells by establishing a memory-like functional state termed trained immunity [3]. The initial challenge induces stable chromatin remodeling, changes in DNA methylation, and shifts in cellular metabolism that collectively “prime” innate cells for heightened responses to subsequent stimuli. As such, innate memory is antigen-agnostic and stimulus-shaped (i.e., different triggers imprint different response programs), whereas adaptive memory is antigen-specific [6].

Overall, trained immunity is a functional reprogramming of the innate immune cells driven by long-term epigenetic and metabolic alterations, resulting in enhanced responses upon subsequent exposure to pathogens [7]. Initially observed following Bacillus Calmette–Guérin (BCG) vaccination [8] and β-glucan stimulation [9], trained immunity is now recognized as a central aspect of immune plasticity. From a mechanistic point of view, a central feature is epigenetic remodeling, including histone modifications that change how easily inflammatory/antimicrobial genes can be transcribed upon restimulation, including (i) histone acetylation (e.g., increased histone H3 lysine 27 acetylation (H3K27ac) at enhancers) to open chromatin and support rapid transcription, and (ii) histone methylation patterns (commonly involving increased activating marks such as histone H3 lysine 4 trimethylation (H3K4me3) at promoters) to create a “poised” state [6,10].

Epigenetically, trained immunity is characterized by stimulus-dependent chromatin priming, including activating histone modifications and enhancer reconfiguration, which place inflammatory and antimicrobial genes in a poised state and enable faster and stronger transcription upon restimulation [6,10]. Metabolically, trained cells undergo sustained rewiring involving increased glycolysis, mechanistic target of rapamycin (mTOR) signaling, and alterations in the tricarboxylic acid (TCA) cycle and cholesterol pathways, generating metabolites that directly influence chromatin-modifying enzymes [11,12]. Enhancer reconfiguration can generate latent enhancers that become active only after the first stimulus, enabling faster/broader responses later [13].

Although it could be advantageous in infectious contexts [14], this reprogramming within the context of chronic inflammatory and/or autoimmune diseases (ADs) could trigger faster inflammatory responses, amplify antigen presentation, and sustain pro-inflammatory pathways, thus favoring disease initiation or propagation.

## 2. Trained Immunity in ADs

ADs arise when the immune system mounts an aberrant response that misidentifies healthy tissues as foreign and attacks them accordingly. Most ADs are chronic and lack a definitive cure, although their symptoms can be managed through appropriate therapeutic interventions [15]. Current understanding positions the innate immune system at both the initiation and culmination of pathophysiologic processes in autoimmunity [16]. The breakdown of self-tolerance that triggers autoimmunity is thought to originate largely from innate immune cell activity [16]. Likewise, the ensuing inflammatory cascade and immune-mediated tissue damage within the target organ are driven predominantly by innate immune cells and their effector molecules, while adaptive immunity plays a more selective role, shaping the anatomical and organ specificity of the autoimmune response [16,17]. As such, innate immune cell priming might be a key modulator of disease initiation and progression [16]. Furthermore, the effects of the initial stimuli are highly dose-dependent: for instance, low-dose stimulation of monocytes with lipopolysaccharides (LPS) or flagellin has been shown to induce trained immunity, whereas higher doses of these stimuli elicit the opposite effect, namely immune tolerance [18].

In this Perspective, we propose a conceptual framework in which trained immunity functions as a context-, time-, and tissue-dependent regulator of innate immune responsiveness. Within this framework, identical training stimuli can exert protective, pathogenic, or therapeutically exploitable effects depending on (i) the inflammatory context in which training occurs, (ii) the timing relative to disease onset and progression, and (iii) whether trained immune programs are confined to tissue-resident cells or systemically imprinted at the level of circulating myeloid cells or hematopoietic progenitors.

**Figure 1:**
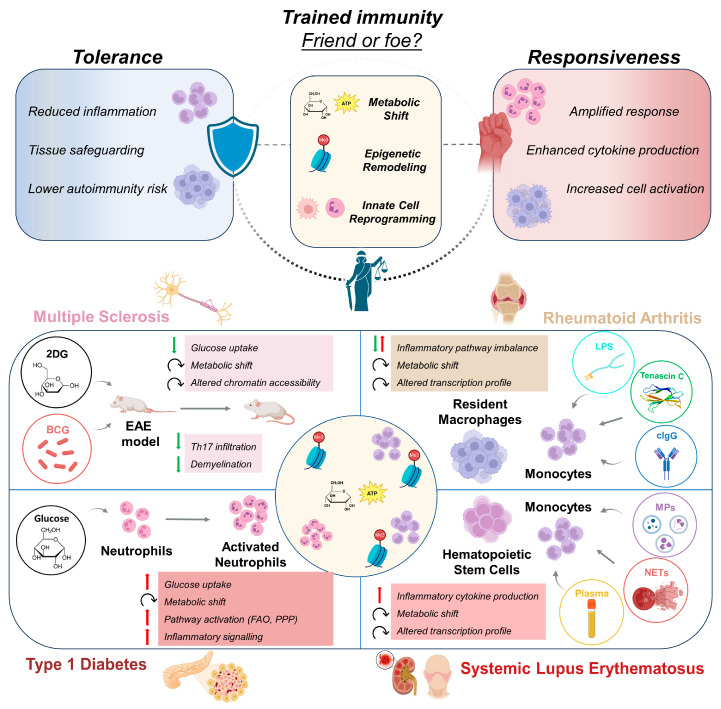
Trained immunity’s central role in immune plasticity. Initial triggering of the immune response by viral particles and bacteria or its components (proteins, sugars, etc.) functionally reprograms innate immune cells towards a more responsive pro-inflammatory phenotype or a tolerant anti-inflammatory profile. Mechanistically, trained immunity is sustained through long-term epigenetic (responsive: H3K4me3, H3K27ac; tolerant: H3K9me3, H3K27me3) and metabolic alterations (responsive: increased glycolysis and mevalonate pathway; tolerant: increased oxidative phosphorylation and fatty acid oxidation), which can result in altered responses upon subsequent exposure to a broad range of pathogens. While such reprogramming may be beneficial during infections, in the setting of chronic inflammatory and/or ADs, such as MS, RA, T1D, and SLE, it could instead accelerate inflammatory responses, enhance antigen presentation, and perpetuate pro-inflammatory pathways, thereby promoting disease onset or progression. Created with Biorender.com.

Using this framework, we examined how trained immunity contributes to AD pathophysiology across distinct clinical settings. Specifically, we discuss multiple sclerosis (MS), rheumatoid arthritis (RA), type 1 diabetes (T1D), and systemic lupus erythematosus (SLE) as representative examples of organ-specific and systemic autoimmunity (Figure 1), while also referencing emerging evidence in Sjögren syndrome [19], vitiligo [20], and psoriasis [21]. Rather than cataloging mechanisms in isolation, each disease is interpreted in terms of the spatial distribution, durability, and functional consequences of trained innate immune programs.

### 2.1. Multiple Sclerosis

MS is a chronic disease characterized by immune-mediated attack on the central nervous system (CNS), resulting in the destruction of myelin [22]. This damage arises from the infiltration of lymphocytes and macrophages into the CNS, where their activity contributes to demyelination and, if left unresolved, can progress to neurodegeneration [22]. Notably, the CNS retains an intrinsic capacity for repair through remyelination, a process that is also modulated by lymphocytes and macrophages [23]. Within this framework, macrophages exhibit functional plasticity, where they may assume a pro-inflammatory phenotype that amplifies myelin injury or a pro-regenerative phenotype supporting remyelination [22]. Spatially, trained immunity in MS appears to be largely confined to the CNS, affecting resident microglia and infiltrating macrophages that are repeatedly exposed to local inflammatory cues [24]. In terms of durability, recurrent inflammatory stimulation, together with genetic susceptibility, promotes sustained metabolic and epigenetic reprogramming of these innate cells, allowing trained immune features to persist over time. Functionally, this persistent lowering of activation thresholds may bias innate immune cells toward exaggerated pro-inflammatory responses, thereby amplifying demyelination and neurodegeneration while potentially limiting regenerative processes such as remyelination.

Studies using the experimental autoimmune encephalomyelitis (EAE) mouse model of MS suggest a potential role for trained immunity in disease pathogenesis, potentially mediated by toll-like receptor (TLR) 9 [25]. More recently, it has been reported that both helminth products [26] and Type A CpG oligodeoxynucleotides [27] exert a protective effect over the development of the disease, which further supports the link between trained immunity and MS pathogenesis through TLR9 signaling [26,27]. The BCG vaccine has also been shown to enhance innate immune responses by stimulating TLRs, particularly TLR2 and TLR4, which are key in immune activation [28]. In EAE, BCG administration alleviates symptoms by reducing the number of T helper (Th)-17 cells in CNS and demyelinated lesions [29]. However, this effect appears to involve adaptive immunity more than innate mechanisms. 

Another study revealed through untargeted metabolomics analysis that peripheral blood mononuclear cells (PBMCs) from relapsing–remitting MS (RRMS) patients displayed heightened glycolysis compared with those from healthy controls [30]. They further studied the role of glycolysis and its modulation of disease progression in the mouse model, by treating EAE mice with 2-deoxy-D-glucose (2DG), a known glycolytic inhibitor, where they observed a lower glucose uptake and metabolic reprogramming of monocytes/macrophages, leading to delayed disease onset and reduced severity [30]. Epigenetic regulation in MS has been the focus of recent studies, where regions of open chromatin in monocytes and genetic loci associated with MS were shown to be linked. In particular, MS-associated variants can influence key immune regulatory genes, including Nuclear Factor Kappa B Subunit 1 (*NFKB1*), Signal transducer and activator of transcription 3 (*STAT3*), and Interferon Regulatory Factor 8 (*IRF8*) [31]. Furthermore, altered chromatin accessibility in these cell populations may increase their responsiveness to environmental stimuli, promoting epigenetic reprogramming and the amplification of pro-inflammatory activity, indicating that genetic risk variants modulating gene expression in microglia may contribute to MS through dysregulated immune responses [32]. In light of the available evidence, trained immunity may already be an intrinsic feature of MS pathogenesis, and its modulation could therefore represent a therapeutic avenue [32].

### 2.2. Rheumatoid Arthritis

RA is among the most common autoimmune diseases globally, affecting approximately one percent of the population, and is marked by systemic immune dysregulation and altered calcium signaling that predominantly involves the synovial joints [33]. Trained immunity in RA involves both synovium-resident macrophages and circulating monocytes that are continuously exposed to endogenous inflammatory stimuli within joint tissues and the systemic compartment [34]. Persistent exposure to autoantibodies, immune complexes, and extracellular-matrix-derived ligands promote sustained metabolic and epigenetic reprogramming, lowering activation thresholds over prolonged periods [35,36]. This persistent trained state may reinforce synovial inflammation, enhances crosstalk with stromal cells, and sustains joint-specific tissue damage, thereby driving disease progression and chronicity [37]. Using transcriptomic and epigenetic profiling of monocytes from healthy individuals and RA patients, as well as macrophages from RA synovium, researchers found that tenascin-C uniquely reprograms monocytes [38]. Priming with tenascin-C generated both heightened and suppressed transcriptional responses upon later stimulation. Although tenascin-C and LPS share some downstream gene signatures, each induces distinct transcriptional programs driven by stimulus-specific epigenetic mechanisms [38]. In healthy monocytes, tenascin-C priming produced trained responses to stimuli abundant in RA joints, including activation of genes linked to chronic inflammation, tissue degradation, metabolic dysfunction, and poor therapeutic outcomes, patterns not induced by LPS [38]. Many of these genes were already elevated in RA monocytes and synovial macrophage subsets associated with disease flare, accompanied by permissive and bivalent epigenetic marks [38]. These findings demonstrate how endogenous triggers drive persistent inflammatory programming and identify pathways for targeted intervention without broad immunosuppression [38].

Furthermore, RA-associated autoantibody deposits can prime human monocytes to exhibit an exaggerated inflammatory response, especially through heightened production of tumor necrosis factor (TNF)-α [39]. Comparative transcriptomic profiling of cells trained with plate-bound human IgG (cIgG) versus β-glucan revealed that a glycolytic metabolic shift is a central driver of this trained phenotype [39]. Gene signatures induced by cIgG were enriched in synovial tissues from individuals with anticitrullinated protein antibody (ACPA)-positive arthralgia, undifferentiated arthritis, early RA, and established RA, closely resembling the myeloid pathotype and suggesting prior in vivo priming events [39]. Thus, RA-specific autoantibodies can train monocytes within inflamed tissues from the asymptomatic phase, offering new insights into disease progression and potential avenues for targeted therapeutic or preventive intervention [39].

In RA, tenascin-C and immune complexes are abundantly present in synovial tissue and circulation, making it difficult to experimentally distinguish long-lived reprogramming from ongoing stimulation [40,41]. If monocytes exposed to these endogenous ligands acquire a durable, autonomous, hyper-responsive phenotype that remains detectable after a resting phase, this would strongly support the existence of trained immunity in RA. On the other hand, if their inflammatory phenotype rapidly wanes once these signals are removed, the process would be better interpreted as sustained priming rather than innate immune memory [42]. As such, more evidence regarding the long-term effects of tenascin-C and immune complexes on monocytes upon inhibition or removal of these molecules is needed.

Recently, it was demonstrated that β-glucan-induced trained immunity worsens inflammatory arthritis by suppressing ferroptosis in synovial fibroblasts through IL-1β-mediated macrophage–synovial fibroblast crosstalk in the collagen-induced arthritis (CIA) model [43]. The authors demonstrate that N-acetyltransferase 10 (NAT10)-dependent N4-acetylcytidine (ac4C) modification of Ferroptosis Suppressor Protein 1 (FSP1) mRNA drives ferroptosis resistance, leading to immune dysregulation and joint damage [43]. This work, as well as the previously cited articles, suggests that trained immunity features might be responsible for RA progression through, for example, dampening ferroptosis, although some of these findings require validation in humans. Interestingly, given the aforementioned evidence that sustains the impact of trained cells and their byproducts, such as autoantibodies, on synovium-resident cells, and consequently on the pathogenesis of the disease, pharmacologically targeting trained immunity might represent a valid adjuvant therapy for RA.

### 2.3. Type 1 Diabetes

T1D is a chronic disease characterized by immune cell infiltration in the pancreatic islets that ultimately leads to the destruction of β cells [44]. In particular, neutrophils in T1D are predisposed to excessive NET formation, a process that impairs wound healing in affected patients [45]. Trained immunity in T1D primarily affects circulating innate immune cells, particularly neutrophils [46], with downstream consequences for pancreatic islets and peripheral tissues. Chronic hyperglycemia induces sustained metabolic and epigenetic reprogramming that persists beyond acute glucose fluctuations, imprinting long-lasting inflammatory memory [47]. Thus, this trained phenotype would amplify NET formation [48] and cytokine production, thereby contributing to β cell destruction and systemic inflammatory complications [6]. 

Shrestha et al. demonstrated that trained immunity underlies diabetes-associated NET priming [45]. Hyperglycemia functions as a bona fide training stimulus for neutrophils that undergo metabolic reprogramming characterized by increased glycolysis, activation of the pentose phosphate pathway (PPP), and enhanced fatty acid oxidation (FAO), collectively driving the accumulation of acetyl-coenzyme A (acetyl-CoA) [45]. Elevated acetyl-CoA generated through ATP-citrate lyase (ACLY), together with histone acetyltransferase activity, promotes acetylation of histone 3 (H3K9, H3K14, H3K27) and histone 4 (H4K8). Pharmacologic inhibition of these enzymes fully abrogates high-glucose-induced NET priming. Neutrophils from patients with T1D exhibited the same trained phenotype, confirming that trained immunity contributes to functional rewiring in vivo [45].

These findings delineate a mechanistic framework in which hyperglycemia reconfigures neutrophil metabolism, by stimulating glycolysis, PPP, and FAO, to generate acetyl-CoA that drives ACLY-dependent histone acetylation [45]. This epigenetic reprogramming establishes trained immunity and amplifies NET formation and cytokine production, perpetuating chronic inflammation and impaired tissue repair. The ACLY–acetyl-CoA–histone acetylation axis therefore represents a compelling therapeutic target for mitigating inflammatory complications and defective wound healing in diabetic patients.

Moreover, hyperglycemia contributes to vascular complications typically associated with diabetes, such as atherosclerosis, myocardial infarction and diabetic kidney disease, by secreting pro-inflammatory cytokines and promoting chronic inflammation [49]. Trained immunity has a significant impact on the rise in these comorbidities since hyperglycemia was shown to be responsible for priming monocytes and hematopoietic stem cells (HSCs), leading to a prolonged pro-inflammatory profile [50]; since chronic inflammation during pregnancy is known to increase the offspring’s risk for cardiovascular [51] and metabolic diseases [52], trained immunity might be an intrinsic part of a cycle, interconnecting chronic inflammation and generational increased risk of developing T1D. As such, it becomes increasingly important to deepen the knowledge on the impact of trained immunity in the pathophysiology of metabolic autoimmune diseases and consider targeting “trained” features for treating T1D. 

### 2.4. Systemic Lupus Erythematosus

SLE is an autoimmune disorder characterized by immune responses against nuclear antigens, including those originating from apoptotic microparticles (MPs) and NETs [53]. Trained immunity in SLE is systemically distributed, involving circulating monocytes and other innate immune populations exposed to nuclear antigens across multiple organs [54]. Repeated exposure to endogenous danger signals and immune complexes sustains epigenetic and metabolic reprogramming over time. This persistent lowering of activation thresholds may promote exaggerated cytokine responses, multi-organ inflammation, and relapsing disease activity characteristic of SLE [55]. 

A study by Yanginlar et al. examined whether such nuclear autoantigens can elicit trained immunity in SLE [54]. Upon exposure to SLE-associated nuclear antigens, NETs, MPs, or plasma from SLE patients and following a five-day resting period, purified monocytes were restimulated with TLR agonists, and cytokine production was quantified by enzyme-linked immunosorbent assay (ELISA). The authors demonstrated that MPs, NETs and SLE patient plasma are able to induce a trained immunity phenotype in vitro [54]. Consistently, circulating monocytes from individuals with SLE exhibited increased pro-inflammatory cytokine responses to TLR ligands, reflective of trained immunity [54]. These functional changes were accompanied by dysregulated H3K4me3 patterns and increased expression of genes linked to metabolic activation and inflammation, demonstrating that nuclear antigens can drive trained immunity, which contributes to the broader immune dysregulation observed in SLE [54]. However, it remains unclear if the trained immunity features in innate immune cells of SLE patients can be reversed and thus become a therapeutic target for a focused or adjuvant therapy. Considering that SLE management is guided by a treat-to-target strategy focused on reaching and maintaining remission or low disease activity [56], rewiring the metabolism and epigenetic marks of trained cells could contribute to achieving a stable remission state.

## 3. Can Trained Immunity Be Modulated?

Trained immunity is highly sensitive to the exposome, that is, the cumulative measure of environmental exposures encountered across the life course [57]. Microbial [58], dietary [59], metabolic [60], and lifestyle [61] factors represent potent modulators of innate immune training through impacts on cellular metabolism, epigenetic landscapes, and inflammatory tone.

It can be modulated at several levels: induction, maintenance, and the effector phase [62]. All of these are integrated through environmental exposures, metabolic cues, epigenetic regulation, and cytokine signaling, offering multiple opportunities for therapeutic intervention [60].

At the inductive level, trained immunity is molded by microbial products, vaccination, infection, and endogenous danger signals [60]. Anti-inflammatory cytokines such as IL-10 can interfere with training by inducing tolerance and limiting the establishment of inflammatory innate immune programs [63]. Genetic variation and timing of exposure further influence the magnitude and durability of trained immune responses [64]. These mechanisms ensure the persistence of trained immunity at the maintenance level through metabolic and epigenetic mechanisms. The key metabolic pathways include glycolysis, mitochondrial metabolism, and cholesterol biosynthesis, which further support epigenetic modifications including histone methylation, acetylation, and lactylation [65]. Targeting metabolic enzymes or epigenetic writers/readers pharmacologically, along with upstream inflammatory mediators, can reset maladaptive innate immune memory without fully suppressing immune competence [66]. This form of trained immunity thus manifests clinically at the level of effector function as enhanced production of cytokines, reactive oxygen species generation, and antimicrobial activity [60]. These outputs can be selectively restrained by regulatory cytokines [67], lifestyle factors [61], and environmental exposures [68], allowing for fine-tuning of the inflammatory response to retain host defense [60,66]. Together, these mechanisms position trained immunity as a tunable immunological process. Strategic modulation, either attenuation in autoimmune and chronic inflammatory diseases or enhancement in settings of infection susceptibility, represents a promising avenue for precision immunotherapy [69].

The fine-tuning of trained immunity induction is crucial to adapt this strategy to a broad range of diseases. At a conceptual level, pathologies that intrinsically feature a hyperactivated pro-inflammatory immune system, such as neuroinflammatory, chronic inflammatory and ADs, may be counteracted through the suppression of epigenetic features associated with trained immunity; on the other hand, cancer, frequent reinfections, and poor response to vaccines could benefit from triggering an exacerbated trained immune response. 

As previously introduced, different approaches are currently used to elicit a faster and more intense immune response, including: (i) the BCG vaccine, which can reduce the impact of a broad range of infections and is able to train hematopoietic stem cells [70]; (ii) PRR ligands, such as β-glucan and TLR agonists (for example, Pam3CysSerLys4 (Pam3CSK4) and LPS), which can induce a more controlled trained immunity effect [71]; (iii) other molecules like Oxidized Low-density Lipoprotein (oxLDL), which can bind multiple receptors and induce a long-lasting metabolic shift [60], by acting on the mevalonate–mTOR–hypoxia inducible factor (HIF)-1α axis and promoting epigenetic reprogramming, particularly H3K4me3 and H3K27ac at promotors and enhancers of pro-inflammatory genes on mature monocytes [70].

Moreover, the presence of trained immunity features can serve as a biomarker for the risk of developing an AD, given that innate immune overactivation is generally an early step associated with autoimmunity [72]. Particularly, epigenetic marks in the promotors and enhancers of genes that codify pro-inflammatory cytokines, such as TNF-α, IL-6 and IL-1β, ensure a persistent inflammatory environment and an amplification of immune responses, which sustain hyperactive autoreactive cell survival [6,10,73]. As such, the hypothesis that therapies targeting adaptive immune cells might be less effective in autoimmune disease patients with a trained phenotype arises.

Beyond ADs, dysregulated innate immune responses also play a central role in autoinflammatory diseases, where chronic inflammation arises primarily from aberrant activation of the innate immune system rather than antigen-specific adaptive immunity [74]. Current knowledge indicates that Adult-onset Still’s disease (AOSD) may involve mechanisms of innate immune reprogramming akin to trained immunity that contribute to persistent inflammatory responses, though direct evidence in humans is currently lacking. This is because AOSD is driven by dysregulated innate immunity and can be triggered by viral infections, which may induce trained immunity features [75]. Moreover, it might contribute to macrophage activation syndrome (MAS), which is a common comorbidity in autoinflammatory diseases where there is a hyperactivation mainly of macrophages that severely worsens the systemic inflammation [76].

Trained immunity features are reversible. In particular, the metabolic shift towards glycolysis or the mevalonate pathways is highly dependent on the availability of nutrients and it can be blocked by commonly available drugs, such as rapamycin or metformin, that inhibit mTOR and activate 5’ adenosine monophosphate-activated protein kinase (AMPK) respectively, preventing or reverting the metabolic shift typical of a trained phenotype, thus restoring cell response intensity and blocking trained immunity, as demonstrated in vitro [77] and in animal models [78]. 

**Figure 2:**
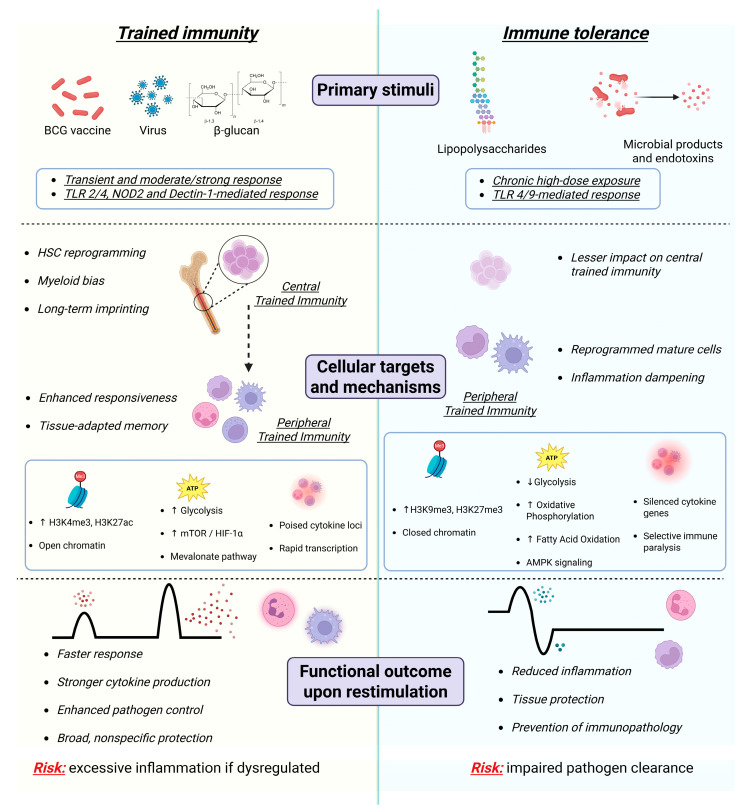
Trained immunity versus immune tolerance: stimuli, mechanisms, and functional outcomes. Schematic comparison of trained immunity (**left**) and immune tolerance (**right**) highlighting differences in primary stimuli, cellular targets, molecular mechanisms, and functional consequences upon restimulation. Trained immunity is induced by transient or moderate inflammatory stimuli such as BCG vaccination, viral infections, or β-glucan, primarily through TLR2/4, Nucleotide-binding oligomerization domain-containing protein 2 (NOD2), and Dectin-1 signaling. It involves both central reprogramming of HSCs, leading to myeloid bias and long-term imprinting, and peripheral reprogramming of mature innate immune cells, resulting in enhanced responsiveness and tissue-adapted memory. These processes are supported by epigenetic changes associated with open chromatin (e.g., increased H3K4me3 and H3K27ac), metabolic rewiring toward glycolysis and the mTOR/HIF-1α and mevalonate pathways, and poised cytokine loci that enable rapid transcription. Functionally, trained immunity leads to faster and stronger cytokine responses, improved pathogen control, and broad, nonspecific protection, but carries the risk of excessive inflammation if dysregulated. Created with Biorender.com.

On the other hand, epigenetic marks are more persistent, but gradually decay over time in the absence of stimuli, both because innate immune cells are typically short-lived and due to the active process of chromatin remodeling that might remove or modify trained immunity marks [79]. However, when HSCs are involved, their constant differentiation into innate immune cells ensures the epigenetic signature remains present in circulating “trained” cells, conferring a trained immune phenotype potentially lasting several years [7,14,80] (Figure 2).

In contrast, immune tolerance arises from chronic or high-dose exposure to microbial products such as lipopolysaccharides and endotoxins, mainly via TLR4/9 signaling [81]. Tolerance predominantly affects peripheral mature innate immune cells, with a lesser impact on central trained immunity [82], and is characterized by epigenetic repression (e.g., increased H3K9me3 and H3K27me3, closed chromatin [83]), a metabolic shift toward oxidative phosphorylation, fatty acid oxidation, and AMPK signaling [84], and silencing of pro-inflammatory cytokine genes [84,85]. Upon restimulation, tolerogenic cells display reduced inflammatory responses, promoting tissue protection and prevention of immunopathology, at the potential cost of impaired pathogen clearance [82,85]. 

From a theoretical point of view, most immunosuppressive treatments are designed to inhibit immune cell activation, proliferation, or cytokine signaling at the transcriptional or post-transcriptional level [86]. However, these approaches do not reverse the underlying epigenetic modifications, such as increased chromatin accessibility and activating histone marks at pro-inflammatory gene loci, that define trained innate immune cells [69]. As a result, once pharmacological suppression is reduced or withdrawn, these cells can rapidly reinitiate inflammatory transcriptional programs, leading to disease persistence or relapse [87]. Moreover, trained innate immune cells can be reactivated by nonspecific environmental or endogenous danger signals, including tissue-damage-associated molecular patterns generated by ongoing inflammation [88]. This would establish a self-perpetuating inflammatory loop in which inflammation itself reinforces innate immune training, thereby sustaining pathological immune activity despite suppression of adaptive immune responses [89]. On the other hand, readily available drugs can also be used to downmodulate the epigenetic marks typical of a trained signature; in particular, inhibitors of histone methyltransferases, deacetylases and bromodomain (BET) may control exacerbated inflammation, although in vivo they act in a nonspecific manner, which might lead to general immunosuppression [90].

## 4. Conclusions and Future Directions

Although the protective role of trained immunity against infections and its contribution to vaccine efficacy has been widely appreciated, its relevance in ADs is increasingly being recognized. Autoimmunity has traditionally been regarded as a maladaptive consequence of a dysregulated adaptive immunity [15]. However, recent mounting evidence has placed innate immune memory as an important upstream driver of chronic inflammation and tissue damage [10]. This has raised the question of whether trained immunity acts as a friend that enhances host defense, a foe that fuels autoimmunity, or a therapeutic target that can be modulated for clinical benefit.

Trained immunity likely evolved as an adaptive advantage, enabling rapid and robust responses to recurrent infections [14]. Considering this strong innate response as beneficial in an autoimmunity context, the advantage of doing so may be depicted in the susceptibility of several ADs to infections through either immune dysregulation or immunosuppressive therapies. Trained immunity could, theoretically, add that layer of protection that compensates for adaptive immune defects. Moreover, early-life microbial exposures which induce trained immunity were associated with reduced risk of immune-mediated diseases [91], supporting the hygiene hypothesis, which posits a protective influence of microbial exposure in early life on the development of allergy and asthma and originally emphasized adaptive immune dysregulation, particularly Th1/Th2 imbalance [92].

Indeed, growing evidence suggests that trained immunity plays a significant role in the pathogenesis and perpetuation of ADs. Training stimuli include endogenous damage-associated molecular patterns (DAMPs) [93], oxidized lipids [94], and chronic exposure to inflammatory cytokines [65,95], among others. Unlike transient microbial exposure, these signals are sustained, leading to long-term hyper-responsiveness of innate cells. This maladaptive training can enhance tissue inflammation, increase antigen presentation, and lower the threshold for adaptive immune activation [96]. Rather than passively supporting autoimmunity, trained immunity may itself be a primary driver, establishing a self-sustaining inflammatory loop, relatively impervious to classical immunosuppressive therapies. The hypothesis that the exacerbated immune response elicited by trained immunity can lead to the development of ADs may represent a valuable research hot topic in the near future, potentially clarifying the etiology of poorly understood pathologies [97].

Trained immunity appears to contribute to both organ-specific and organ-nonspecific ADs, although its characteristics differ substantially between these two settings. In organ-specific autoimmunity, trained immunity is largely confined to tissue-resident innate immune cells, such as microglia in MS or macrophages in pancreatic islets in T1D. These cells acquire long-lasting epigenetic and metabolic changes that enhance their responsiveness to local stimuli, thereby sustaining inflammation within a restricted anatomical site [98,99].

In contrast, in organ-nonspecific ADs, trained immunity is more systemic in nature. Circulating monocytes, neutrophils, and even bone marrow progenitor cells undergo functional reprogramming, leading to a persistent pro-inflammatory phenotype that can affect multiple organs simultaneously [100]. This systemic imprinting may explain the chronic, relapsing, and multi-organ manifestations observed in diseases such as SLE and RA [101].

Thus, while trained immunity represents a unifying mechanism linking innate immune memory to autoimmunity, its spatial distribution, cellular targets, and pathological consequences differ between organ-specific and organ-nonspecific diseases. Acknowledging these differences may be critical for designing therapeutic strategies that selectively modulate trained immunity in a tissue-specific or systemic manner.

Recognizing the role of trained immunity in autoimmunity opens new possibilities for therapeutic interventions. This contrasts with adaptive immune memory, which is not readily reversible. Trained immunity transcends the traditional dichotomy between innate and adaptive immunity, reshaping our understanding of autoimmune disease mechanisms. Neither purely friend nor foe, its impact depends on timing, context, and duration of activation [102].

Context should be defined in terms of the underlying disease setting and immune baseline, as the same trained immunity stimulus may be beneficial in infectious diseases and vaccination, but detrimental in cancer or chronic inflammatory disorders [7]. Chronic low-grade inflammation is an also central pathogenic driver of aging-associated diseases, including cardiovascular disease, neurodegenerative disorders, metabolic syndrome, and cancer [103]. In neurodegenerative disease, systemic inflammation can “train” microglia to produce excessive pro-inflammatory cytokines upon restimulation, promoting persistent neuroinflammation and tissue damage. In this setting, trained immunity amplifies pathology and fuels disease progression [104]. In tumors, trained immunity has a dual role because it amplifies innate immune responses in a way that can either enhance anti-tumor immunity or promote tumor progression, depending on context [105]. As a benefit, trained immunity can strengthen anti-tumor defense. Reprogrammed monocytes, macrophages, and natural killer (NK) cells display increased cytokine production, antigen presentation, and cytotoxic activity. This can enhance tumor recognition, promote T cell activation, and improve immune-mediated tumor clearance [106]. For example, BCG-induced trained immunity is therapeutically exploited in bladder cancer, where it activates innate immune cells that contribute to tumor destruction. In this setting, trained immunity acts as a friend by boosting immune surveillance and reinforcing anti-cancer immunity [107]. As a detriment, trained immunity can also sustain chronic inflammation within the tumor microenvironment. Persistently activated innate immune cells may produce pro-inflammatory cytokines, growth factors, and angiogenic mediators that support tumor growth, tissue remodeling, immune suppression, and metastasis [108]. Trained macrophages can acquire tumor-promoting phenotypes that enhance immune evasion, inhibit cytotoxic T cell function, and foster a pro-tumor niche [109]. Here, trained immunity becomes a foe by maintaining an inflammatory environment that tumors can exploit. Altogether, current evidence indicates that induction of trained immunity prior to antigen exposure or infection could enhance protective immune responses, whereas induction in an already inflamed or autoimmune environment may amplify tissue-damaging inflammation. Experimentally, this requires comparative studies in which identical training stimuli are applied across distinct disease models to directly assess context-dependent outcomes. 

Timing represents a second critical determinant and can be operationalized by distinguishing preventive, acute, and chronic phases of disease. Induction of trained immunity before disease onset or early during infection may improve pathogen clearance and immune responsiveness, while induction during chronic inflammation may prolong or exacerbate pathology [10]. This generates testable hypotheses such as whether early training improves resolution of infection whereas late training sustains inflammatory circuits. Longitudinal experimental designs and time-controlled interventions in animal models, together with serial sampling in clinical cohorts, are necessary to define these temporal windows.

Duration refers to the persistence and reversibility of trained immunity. Short-term functional reprogramming driven by metabolic changes may be beneficial and controllable, whereas long-lasting epigenetic imprinting may carry a higher risk of sustained immune dysregulation [96]. This suggests that transient metabolic interventions could be prioritized as safer and more flexible therapeutic tools, while epigenetic therapies, although potentially powerful, require careful evaluation due to their durability and lack of cell-type specificity [110]. A key hypothesis is that reversible metabolic modulation can fine-tune inflammatory responses without inducing long-term pathogenic memory, in contrast to epigenetic interventions that may generate persistent immune phenotypes. Together, these considerations define concrete research priorities. 

Standardized models are needed in which training stimulus, dose, timing, and duration are systematically varied, combined with single-cell epigenomic and metabolic profiling to track the stability of trained states [111]. Clinically, patient stratification based on inflammatory burden, infection risk, and disease stage should precede any attempt to modulate trained immunity. Early translational efforts should focus on reversible metabolic modulators, reserving epigenetic therapies for severe or refractory conditions under strict monitoring. This framework converts the conceptual duality of trained immunity into a set of operational hypotheses and structured experimental and clinical strategies.

Approaches with direct clinical potential benefit from existing clinical experience, defined pharmacokinetics, and established safety profiles in other disease contexts—as in the case of rapamycin and metformin as explored in previous sections—making them attractive candidates for repurposing in the modulation of trained immunity. In contrast, interventions aimed at shaping the exposome, such as dietary modification or manipulation of the microbiome, remain largely exploratory and are likely to exert highly individualized and context-specific effects, which complicates their standardization and clinical implementation. However, all of these strategies carry substantial risks that must be carefully weighed. 

Trained immunity maintains innate immune cells in a hyper-responsive state by strengthening host defense and provides broad, nonspecific protection [80]. This framework challenges prevailing disease models that largely attribute chronic inflammatory and autoimmune disorders to maladaptive adaptive immunity alone. Instead, it positions long-lived innate immune reprogramming as an active and central driver of disease initiation, amplification, and persistence. Thus, trained immunity is highly context-dependent. It is beneficial when it enhances immune surveillance and tumor killing, but detrimental when it promotes chronic inflammation, immune dysregulation, and tumor-supportive pathways. This duality makes trained immunity a promising but complex therapeutic target: it must be carefully directed toward anti-tumor immunity while avoiding the reinforcement of tumor-promoting inflammation.

Conceptually, if trained immunity can bolster defense against pathogens, its broad inhibition may impair host defense mechanisms and increase susceptibility to infections. Epigenetic therapies are intrinsically nonspecific at the cellular level and may produce off-target effects or induce long-lasting and potentially irreversible immune reprogramming. Metabolic interventions, while more readily reversible, can have widespread systemic effects that limit their applicability, particularly in patients with metabolic or cardiovascular comorbidities. Similarly, targeting cytokine pathways entails the danger of disrupting immune homeostasis, either by inducing excessive immunosuppression or by perpetuating chronic inflammatory states. These considerations underscore the need for precise patient stratification and tightly controlled therapeutic modulation of trained immunity.

Overall, with this Perspective we propose a unifying framework in which trained immunity is viewed as a dynamic “set point” of innate immune responsiveness that is shaped by metabolic, epigenetic, and environmental cues. Rather than being intrinsically protective or pathogenic, trained immunity modulates the threshold and magnitude of innate immune activation. When tightly regulated and transient, it enhances host defense, tissue repair, and immune surveillance. When persistent or excessive, it lowers activation thresholds, sustains inflammatory circuits, and promotes chronic immune dysregulation, thereby contributing to autoimmunity and tumor-promoting inflammation. 

Targeting trained immunity may provide a way to reset maladaptive innate immune programs without broadly suppressing immune function. Despite the concerns previously addressed, future strategies could include modulation of metabolic pathways, epigenetic reprogramming, cytokine signaling, or controlled microbial and vaccine-based interventions to recalibrate innate immune memory, although further research on the impact of these approaches both at the cellular and systemic levels is still required. Advances in omics technologies, including single-cell transcriptomics, epigenomics, metabolomics, proteomics, and microbiome profiling, now provide the tools to systematically map the molecular signatures of trained immunity across cell types, tissues, and disease stages [112]. These signatures may serve as biomarkers to identify individuals predisposed to exaggerated inflammatory responses at increased autoimmune risk or to predict treatment response, particularly in patients who respond poorly to therapies targeting adaptive immunity alone. 

Trained immunity lacks antigen specificity and it is shaped by the nature of the initial stimulus, tissue context, and cellular identity, resulting in distinct and potentially targetable innate immune programs. Its stability appears sufficient to sustain chronic inflammation over prolonged periods, yet remains dependent on ongoing environmental and metabolic reinforcement, implying that trained immune states may be attenuated or reprogrammed rather than permanently erased. This balance between persistence and plasticity positions trained immunity as both a contributor to disease chronicity and a promising target for therapeutic intervention. A key challenge consists of defining when and how trained immunity should be restrained versus harnessed, enabling precision approaches that restore immune balance while preserving protective immunity.

Finally, several critical gaps remain in understanding how trained immunity contributes to autoimmunity. It is unclear whether trained immunity acts as a primary driver of disease initiation or mainly amplifies established adaptive immune dysregulation, as longitudinal studies testing its modulation before disease onset are lacking. A second unresolved issue is the relative contribution of metabolic versus epigenetic mechanisms to the persistence of pathogenic trained immunity, leading to the hypothesis that metabolic reprogramming supports reversible inflammatory amplification, whereas stable epigenetic imprinting is required for chronic autoimmune inflammation. The extent to which systemic trained immunity signatures reflect pathogenic programs within affected tissues remains unknown, raising the hypothesis that organ-specific and systemic ADs require distinct therapeutic strategies targeting tissue-resident innate cells versus bone-marrow-derived myeloid populations.

## Data Availability

No new data were created or analyzed in this study. Data sharing is not applicable to this article.
